# Modeling telomere shortening process

**DOI:** 10.1002/qub2.74

**Published:** 2024-12-14

**Authors:** Panpan Han, Yang Zhou, Weihua Deng

**Affiliations:** ^1^ School of Mathematics and Statistics Gansu Key Laboratory of Applied Mathematics and Complex Systems Lanzhou University Lanzhou China

**Keywords:** aging, anomalous diffusion, CTRW, telomere shortening process

## Abstract

Cell senescence has attracted much attention in the long history of human beings, and telomere shortening (TS) is one of the main concerns in the study of cell senescence. To reveal the microscopic mechanism of TS process, we model it based on molecular stochastic process from the perspective of nonequilibrium statistical physics. We associate the TS process with the continuous time random walk and derive the Fokker–Planck equation to describe the length distribution of the TS. We further modify the model describing the TS process, similar to the anomalous tempered diffusion, and derive the Feynman–Kac equation characterizing the functional distribution of the TS process. Finally, we study the statistics related to the critical telomere length *l*_*c*_, including the occupation time and first passage time. These two kinds of statistics help us understand the time scale of cell senescence.

## 1 INTRODUCTION

In recent decades, the problem of population aging has become increasingly serious worldwide. To improve people’s quality of life and extend the average life span, the study of aging turns to be an important and urgent issue. By delving into the mechanism of aging, scientists can better understand how to prevent and treat diseases caused by aging, and then design anti‐aging strategies to improve people’s health.

**FIGURE 1:**
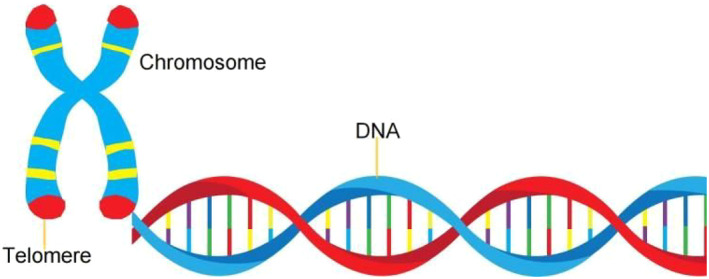
Telomere structure diagram.

Aging is a complex biological process that involves multiple factors, including genes, environment, and lifestyle. By detecting the molecular and cellular changes that occur during aging, one may uncover the potential mechanism and find ways to intervene in aging. The telomere shortening (TS) is one of the main concerns in the study of cell senescence. Telomeres, illustrated in Figure 1, are specific DNA sequences located at the termini of linear chromosomes. Their primary role is to safeguard the structural integrity of chromosomes. Nevertheless, telomeres undergo a gradual shortening process as cells divide and make DNA replication. When telomeres shorten to a critical extent, the stability of the genome within the cell is compromised, ultimately resulting in cell aging, death, or cancer [1]. However, certain specialized cells have the ability to counteract this process through the high expression of telomerase, which can repair and lengthen telomeres [2].

In recent years, there are numerous studies investigating telomeres and telomerase. The discovery of telomeres and predictions regarding their function were made by McClintock and Mill between 1938 and 1941 [3, 4, 5]. In 1961, the concept of the “Hayflick limit” was raised [6]. Subsequently, in 1972, the “telomere end replication problem” of chromosomes was proposed [7], followed by the discovery and measurement of the telomere DNA sequence in the model animal *Tetrahymena* in 1978 [8]. Telomerase was then discovered in 1985 [9]. Later in 1990, it was found that the lifespan of human fibroblasts was associated with TS [10]. Furthermore, in 1998, it was discovered that the overexpression of telomerase reverse transcriptase granted primary human cells with unlimited replication potential [11]. In 1999, the reactivation of human telomerase was found to be crucial for cell differentiation [12]. Finally, in 2009, Elizabeth Blackburn, Carol Greider, and Jack Szostak were awarded the Nobel Prize in Physiology or Medicine for their notable contributions to the understanding of how telomeres and telomerase protect chromosomes. After gaining some understanding of the biological mechanism underlying telomeres and telomerase, several studies have yielded significant findings. Specifically, it has been discovered that TS is implicated in the aging of the bone marrow hematopoietic microenvironment, indirectly impacting the regenerative capacity and differentiation potential of hematopoietic stem cells [13]. The authors in ref. [14] have elucidated the previously unidentified role of the BUB3‐BUB1 complex in the S phase. This research has shed light on how proteins from different pathways collaborate to facilitate the accurate replication and maintenance of telomeres. Moreover, the impact of astragaloside on telomerase activity, telomere length, and the cell cycle of MKN45 human gastric cancer cells was investigated through the employment of telomerase Polymease Chain Reaction ELISA, telomere DNA hybridization, and flow cytometry techniques [15].

There are three primary factors that result in TS. Firstly, the incomplete replication of chromosome ends can lead to the progressive erosion of telomeres. Secondly, the role of exonucleases, enzymes involved in DNA degradation, can also result in telomere loss. Lastly, oxidative stress‐induced damage can further accelerate TS.

In the medical field, the measurement of telomere length has emerged as a valuable diagnostic tool for clinical indications within a hospital setting as demonstrated in ref. [16]. This enables healthcare professionals to make informed care decisions for patients. In the beginning, people observed changes in telomere length by experimental methods and accumulated a lot of experimental data [17]. However, understanding the intricate process of TS at a microscopic level remains challenging due to the highly complex intracellular environment. Specifically, characterizing the precise changes in telomere length before and after cell replication poses difficulties. As a result, researchers have started the journey of investigating the microscopic mechanism underlying the process of TS from a stochastic perspective. The authors in ref. [18] demonstrate that the incomplete replication of chromosome ends is a factor responsible for TS. In this case, the length of the shortened telomere, denoted as *L*_1_, follows a normal distribution. Additionally, the impact of exonuclease activity on chromosomes is another contributor to TS. The probability of exonuclease‐induced TS is tested/assumed to be 0.8, and the length of the shortened telomere, denoted as *L*_2_, adheres to a Poisson distribution. During cell replication, oxidative stress can induce DNA strand damage on telomere, which in turn leads to TS with the tested/assumed probability of 0.1, and the shortened length of *L*_3_ obeys Normal distribution. Finally, the following equation exists:

(1) $$L=1\times \frac{1}{4}\times L_{1}+0.8\times \frac{1}{4}\times L_{2}+0.1\times \frac{1}{2}\times L_{3}\times N,$$

where *L* represents the shortened length of the telomere and *N* is the number of bases damaged in the DNA strand.

In the field of physics, aging refers to the state of a system becoming imbalanced and remaining unstable. A notable example of aging is observed in the behavior of glass [19], which, despite being a solid substance, undergoes structural changes over time, leading to the evolution of its properties. This aging behavior is also observed in the movement of specific proteins in lipid bilayers [20, 21] and in the dynamics of insulin particles within the cytoplasm of living cells [22]. In recent years, scientists have even found aging in the active transport of mRNP molecules along nerve cell microbundles [23].

We hypothesize that the aging of organs can be attributed to molecular processes and their specific behavior within living cells. One possible approach is to connect the biological concept of organ aging to the broader understanding of nonstationary aging at the molecular level. In the intricate environment of a living cell, numerous passive transport processes adhere to the principle of anomalous diffusion [24, 25], as described by the equation. Anomalous diffusion exists at the tissue level [26], although the precise mechanism is not fully understood. The Montroll–Weiss equation can be derived using the continuous time random walk (CTRW) model characterized by two random variables: jump length obeying φ(L) and waiting time abiding by ω(t) [27]

(2) $$\tilde{\hat{p}}\left(k,s\right)=\frac{1-\hat{\omega }\left(s\right)}{s}\frac{1}{1-\tilde{\varphi }\left(k\right)\hat{\omega }\left(s\right)}.$$

Currently, many research findings on telomere and telomerase have been obtained by fitting experimental data to probability distributions. Here, we aim to take a novel approach from the nonequilibrium biology perspective to explore the transformation rule of TS length and its relationship with aging, and to build an effective physical model that can establish connections between the underlying mechanism and the observed distributions and parameters. To achieve these, we will integrate stochastic processes and gene regulation techniques to construct a microscopic dynamic model that accurately depicts TS length. Based on this model, we will derive a macroscopic equation governing the distribution of TS length and consider the associated biostatistics. This methodology represents a crucial step toward quantifying the senescence of cells and organs.

The structure of this paper is as follows. In Section 2, we establish the CTRW model to describe the TS process and derive the corresponding macroscopic equation. Then we calculate the ensemble‐averaged mean square displacement (EAMSD) to explore their properties. In Section 3, the Feynman–Kac equation describing the functional distribution of the TS process is derived from two different perspectives. In Section 4, based on the macroscopic equations obtained in Section 3, biostatistics are discussed, including the distribution of first passage time (the time distribution of TS length first reaching the critical value) and the distribution of occupation time (the residence time of TS length in a specific length interval). In Section 5, this paper summarizes the problems involved and looks forward to future research in this area.

## 2 MODEL

In this section, the CTRW model will be used to conduct microscopic modeling of the TS process, then the Fokker–Planck equation (the probability density function (PDF) *p*(*L*, *t*) satisfies the equation with the TS length *L* at time *t*) will be derived using the Montroll–Weiss equation [28, 29, 30, 31], and the moments that reflect the changing trend of TS length will be calculated.

For building the CTRW model, it is necessary to determine the ω(t) and φ(L). In a living body, the cell cycle changes with the external environment (with a certain randomness), which just happens to correspond to the waiting time, so we choose the time t as the cell cycle and assume that it follows a power law distribution [11, 32]

(3) $$\omega \left(t\right)\;∼\;t^{-\left(1+\alpha \right)}.$$

If 0 < *α* < 1, the mean $T=\int_{0}^{+\infty }t\omega \left(t\right)dt$ diverges; while 1 < *α* < 2, *T* exists. For the choice of the range of *α*, it depends on practical situation. For example, we may choose *α* ∈ (1,2) for infants while choosing *α* ∈ (0, 1) for adults, since telomeres shorten more rapidly in peripheral mononuclear cells of infants than in adults [33]. The waiting time is selected to follow the power law distribution, reflecting the influence of different time scales on telomere length.

**FIGURE 2:**
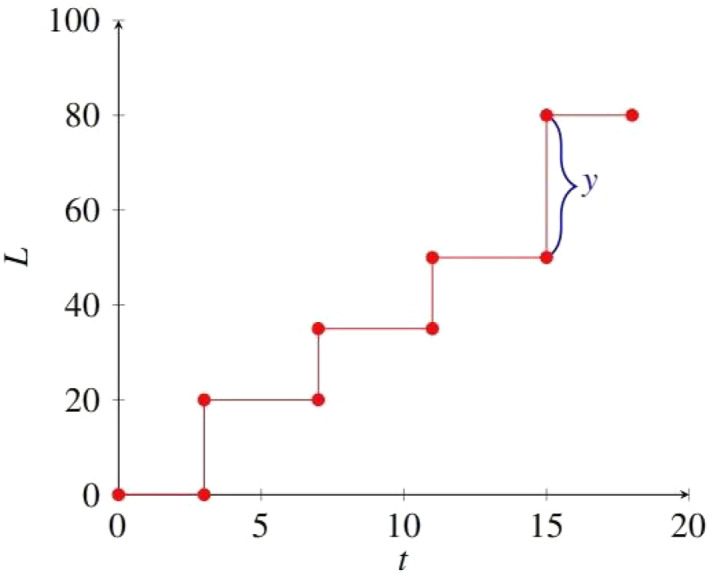
Continuous time random walk model diagram for telomere shortening length. The shortened length *y* is determined only by the incomplete replication of chromosome ends and the action of exonuclease during cellular replication.

The jump length *L* corresponds to TS length, and *L* is assumed to change continuously. Since the probability of DNA strand damage caused by oxidative stress during cell replication is only 0.1, and the probability of TS caused by the action of exonuclease is 0.8, we ignore the effect of oxidative stress here and suppose that exonuclease will cause TS. Then the TS length can be expressed as *L* = *L*_1_ + *L*_2_. Here, we assume that they are independent of each other. Thus, we obtain the following equation:

$$\varphi \left(L\right)=\varphi_{1}\left(L_{1}\right)*\varphi_{2}\left(L_{2}\right)$$

(4) $$∼\sum_{L_{2}=0}^{\infty }\left[\frac{1}{\sqrt{2\pi }\sigma }\exp\;\left\{-\frac{{\left(L-\mu -L_{2}\right)}^{2}}{2\sigma^{2}}\right\}\times \exp\left\{-\lambda \right\}\frac{\lambda^{L_{2}}}{L_{2}!}\right],$$

where “∗” represents the convolution operation, and *λ* is the intensity of the Poisson distribution, taking the value of 155 in the following simulation. *μ* and *σ* are the mean and variance of the normal distribution, respectively, taking values of 60 and 1 in the simulation. Here, the specific values of parameters are chosen according to the experimental results in ref. [18]. At the same time, according to the 3*σ* principle, if the average value is 60, then the value of *L*_1_ has a high probability of being positive, which means that the telomere length will not increase. It can be seen that the selection of parameters is very important for the rationality of the model. To make the correspondence between the research problem and the CTRW model more intuitive, Figure 2 is produced.

When telomeres shorten to a critical extent, the stability of the genome within the cell is compromised, ultimately resulting in cell aging, death, or cancer. In particular, when the length of the shortest telomere in the cell reaches the critical value *l*_*c*_, the cell’s ability to divide will be limited and then gradually senesces. Moreover, telomere length is not infinite at the beginning of cell life, but has an initial length of *l*_0_, so the upper bound of the shortened length *L* is *l*_0_ − *l*_*c*_.

When 0<α<1, the PDF of the waiting time

(5) $$\omega \left(t\right)\;∼\frac{\alpha }{\Gamma \left(1-\alpha \right)}\;t^{-\left(1+\alpha \right)}$$

is with its Laplace transform $\hat{\omega }\left(s\right)∼1-s^{\alpha }$ for small *s* and the asymptotic form of the Fourier transform of *φ*(*L*) in Equation (4) is $\tilde{\varphi }\left(k\right)∼1+ik\left(\lambda +\mu \right)-1/2\sigma^{2}k^{2}$. Substituting the $\hat{\omega }\left(s\right)$ and $\tilde{\varphi }\left(k\right)$ into Equation (2) results in the following:

(6) $$\tilde{\hat{p}}\left(k,s\right)=\frac{1}{s}+\left(\lambda +\mu \right)iks^{-\alpha }\tilde{\hat{p}}\left(k,s\right)-\frac{1}{2}\sigma^{2}k^{2}s^{-\alpha }\tilde{\hat{p}}\left(k,s\right).$$

Next, we verify that the PDF *p*(*L*, *t*) satisfies the normalization, that is, $\int_{-\infty }^{+\infty }p\left(L,t\right)dL=1.$ Here, we use the technique of the Laplace transform. If $\tilde{\hat{p}}\left(k=0,s\right)=\int_{-\infty }^{+\infty }p\left(L,s\right)dL=s^{-1}$, then *p*(*L*, *t*) has normalization. Let *k* = 0 in Equation (6). We have $\tilde{\hat{p}}\left(k=0,s\right)=s^{-1}$, which shows that the derivation process is reasonable. In Equation (6), taking inverse Fourier and Laplace transforms w.r.t. *k* and *s*, respectively, we obtain the following:

(7) ∂p(L,t)∂t=−(λ+μ)∂∂LDt1−α0p(L,t)+12σ2∂2∂L2Dt1−α0p(L,t),

where Dt1−α0p(L,t)=1Γ(α)∂∂t∫0tp(L,τ)(t−τ)1−αdτ is the Riemann–Liouville derivative operator. However, noting that the value range of *L* is [0, *l*_0_ − *l*_*c*_], we set the absorbing boundary condition

(8) $$p\left(L,t\right)=0,L\in \left(-\infty ,0\right)\cup \left(l_{0}-l_{c},+\infty \right)$$

and the initial condition

(9) p(L,0)=δ(L).

When 1<α<2, the power‐law distribution is taken as

(10) $$\omega \left(t\right)∼\alpha \tau_{0}t^{-\left(1+\alpha \right)},$$

where *τ*_0_ is the time scale factor with *τ*_0_ ≪ *t*. The Laplace transform of Equation (10) is $\hat{\omega }\left(s\right)∼1-Ts+Cs^{\alpha }$, *T* = *ατ*_0_/(*α* − 1) is the mean value of the waiting time, and $C=\left|\Gamma \left(1-\alpha \right)\right|\tau_{0}^{\alpha }$ is a constant. Substituting $\hat{\omega }\left(s\right)$ and $\tilde{\varphi }\left(k\right)$ into Equation (2), we have the following equation:

(11) $$s\tilde{\hat{p}}\left(k,s\right)-1=\frac{\left(\lambda +\mu \right)ik}{T}\left(1+\frac{Cs^{\alpha -1}}{T}\right)\tilde{\hat{p}}\left(k,s\right)-\frac{\sigma^{2}k^{2}}{2T}\left(1+\frac{Cs^{\alpha -1}}{T}\right)\tilde{\hat{p}}\left(k,s\right).$$

Similarly, it can also be verified that p(L,t) satisfies the normalization condition. We still apply the inverse Fourier and Laplace transforms on Equation (11). Therefore, the following equation exists:

(12) ∂p(L,t)∂t=−(λ+u)T∂∂L1+CTDtα−10p(L,t)+σ22T∂2∂L21+CTDtα−10p(L,t).

Same as in the previous case, this equation also satisfies the boundary condition (8) and the initial condition (9).

The Equations (7) and (12) are the Fokker–Planck equations (under different assumptions/contexts) that describe the probability distribution of TS length. The *p*(*L*, *t*) can be obtained by solving the partial differential equation with appropriate parameter values combined with boundary conditions, and then the changing trend of TS length distribution can be known through simulation analysis. Note that Equations (7) and (12) are different in form due to the different values of the parameter *α*. In fact, in Equation (12) the two limit cases *C*/*T* ≪ 1 and *C*/*T* ≫ 1 lead to different equations. If *C*/*T* ≪ 1, we have

(13) $$\frac{\partial p\left(L,t\right)}{\partial t}=\frac{-\left(\lambda +\mu \right)}{T}\frac{\partial }{\partial L}p\left(L,t\right)+\frac{\sigma^{2}}{2T}\frac{\partial^{2}}{\partial L^{2}}p\left(L,t\right),$$

which is a standard convection–diffusion equation. If *C*/*T* ≫ 1, we have

(14) ∂p(L,t)∂t=−(λ+μ)T∂∂L+σ22T∂2∂L2CTD0tα−1p(L,t),

which is a time‐fractional convection–diffusion equation.

These results show that the evolution of the TS process is actually between normal and anomalous diffusions, and the essence of this phenomenon is the power law of waiting time distribution *ω*(*t*). As *C*/*T* ≪ 1, the power law of the waiting time disappears, thus leading to the standard convection–diffusion equation.

### 2.1 Moments of each order of the TS length

During the process of cell replication and division, the length of telomeres undergoes changes due to TS. To analyze the trend of the evolution of TS length, we will calculate the EAMSD of TS length. A study simulating telomere dynamics estimates that the average length of TS per cell division is 54 base pairs (bp) [34]. Unlike ref. [34], we will get the average length and variation of EAMSD rather than a number. As before, consider two cases. When 0 < *α* < 1,

$$\langle L(t)\rangle =L^{-1}\left[\left(-i\right)\frac{\partial \tilde{\hat{p}}\left(k,s\right)}{\partial k}{|}_{k=0}\right]$$

$$=L^{-1}\left[\left(\lambda +\mu \right)s^{-\left(1+\alpha \right)}\right]$$

(15) $$=\frac{\lambda +\mu }{\Gamma \left(1+\alpha \right)}t^{\alpha },$$

and the second moment

$$\langle L^{2}\left(t\right)\rangle =L^{-1}\left[-\frac{\partial^{2}\tilde{\hat{p}}\left(k,s\right)}{\partial^{2}k}{|}_{k=0}\right]$$

$$=L^{-1}\left[2{\left(\lambda +\mu \right)}^{2}s^{-\left(1+2\alpha \right)}+\sigma^{2}s^{-\left(1+\alpha \right)}\right]$$

(16) $$=\frac{2{\left(\lambda +\mu \right)}^{2}}{\Gamma \left(1+2\alpha \right)}t^{2\alpha }+\frac{\sigma^{2}}{\Gamma \left(1+\alpha \right)}t^{\alpha }.$$

We use the properties of the Fourier transform $\int_{-\infty }^{+\infty }e^{ikx}x^{n}f\left(x\right)dx={\left(-i\right)}^{n}\frac{d^{n}}{dk^{n}}\tilde{f}\left(k\right)$.

Then we calculate the EAMSD, which is usually a statistic that measures the dispersion of data and tells us how the data points are distributed around the mean. It reflects the stability of TS length and helps us understand its changing trend, and it is expressed as follows:

$$\langle ∆L^{2}\left(t\right)\rangle =\langle L^{2}\left(t\right)\rangle -{\langle L\left(t\right)\rangle }^{2}$$

(17) $$=\left[\frac{2}{\Gamma \left(1+2\alpha \right)}-\frac{1}{\Gamma^{2}\left(1+\alpha \right)}\right]{\left(\lambda +\mu \right)}^{2}t^{2\alpha }+\frac{\sigma^{2}}{\Gamma \left(1+\alpha \right)}t^{\alpha },$$

which has the asymptotic expression

(18) $$\langle ∆L^{2}\left(t\right)\rangle ∼\left[\frac{2}{\Gamma \left(1+2\alpha \right)}-\frac{1}{\Gamma^{2}\left(1+\alpha \right)}\right]{\left(\lambda +\mu \right)}^{2}t^{2\alpha }.$$

**FIGURE 3:**
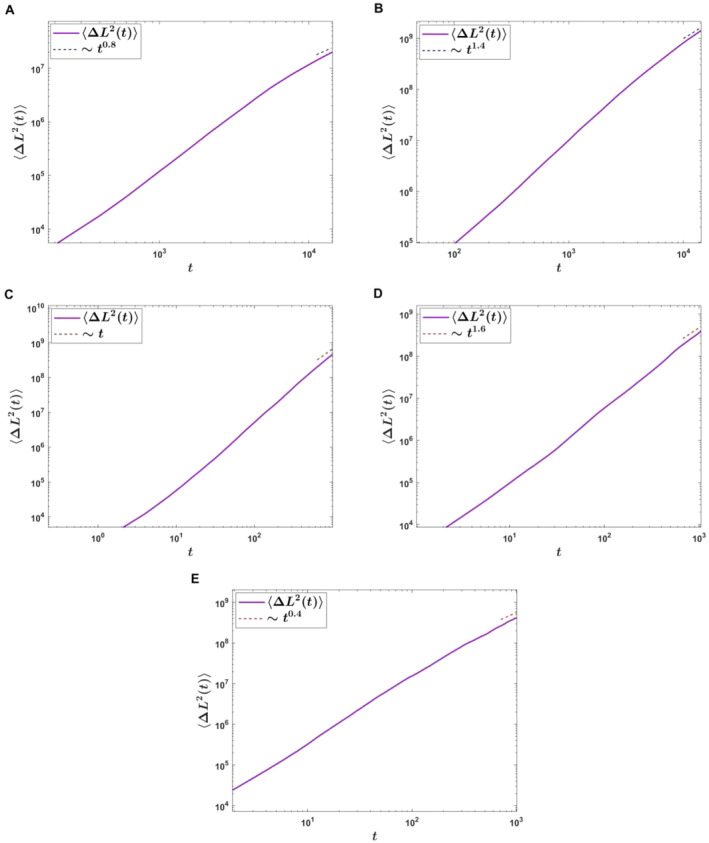
Ensemble‐averaged mean square displacement for the telomere shortening (TS) length. For all the figures, the parameters *λ* = 155, *σ* = 1 and *μ* = 60. Here, the waiting time follows the power‐law distribution (5) with the simulation time *t* = 2 × 10^4^ in panels (A, B), and the waiting time obeys the power‐law distribution (10) in panels (C–E) with the simulation time *t* = 2 × 10^3^. The solid purple line represents the numerical simulation, while the dashed red line is for the analytical results (18) in panels (A, B), and for the analytical results (22) in panels (C–E). In panel (A), *α* = 0.4; in panel (B), *α* = 0.7; in panel (C), *τ*_0_ = 0.01, *α* = 1.1 describing the situation of *C*/*T* ≪ 1; in panel (D), *τ*_0_ = 1, *α* = 1.4; and in panel (E), *τ*_0_ = 1, *α* = 1.8, describing the case of *C*/*T* ≫ 1.

From Equation (18), the TS EAMSD is proportional to *t* ^2*α*^. When *α* ∈ (0, 0.5), the TS process can be regarded as a subdiffusion process. When *α* ∈ (0.5, 1), the TS process can be viewed as a superdiffusion process. In addition, the EAMSD depends on the mean *μ* of the normal distribution and the strength *λ* of the Poisson distribution. Later, the Monte Carlo numerical simulation to the CTRW model will be used to verify the correctness of our calculation results (see Figure 3A,B).

When 1<α<2, the mean is

(19) $$\langle L(t)\rangle =\left\{\begin{array}{ll} \frac{C\left(\lambda +\mu \right)}{T}t, & \text{for}\;\frac{C}{T}\ll 1;\\ \frac{\lambda +\mu }{T}t+\frac{C\left(\lambda +\mu \right)}{T^{2}\Gamma \left(3-\alpha \right)}t^{2-\alpha }, & \text{otherwise};\\ \frac{C\left(\lambda +\mu \right)}{T^{2}\Gamma \left(3-\alpha \right)}t^{2-\alpha }, & \text{for}\;\frac{C}{T}\gg 1, \end{array}\right.$$

and the second moment is

(20) $$\langle L^{2}\left(t\right)\rangle =\left\{\begin{array}{cc} \frac{C\left(\lambda +\mu \right)+\sigma^{2}}{T}t+\frac{C^{2}{\left(\lambda +\mu \right)}^{2}}{T^{2}}t^{2}, & \text{for}\;\frac{C}{T}\ll 1\;;\\ \frac{\sigma^{2}}{T}t+\frac{C\sigma^{2}}{T^{2}\Gamma \left(3-\alpha \right)}t^{2-\alpha }+\frac{2C^{2}{\left(\lambda +\mu \right)}^{2}}{T^{4}\Gamma \left(5-2\alpha \right)}t^{\left(4-2\alpha \right)} & \\ +\frac{4C{\left(\lambda +\mu \right)}^{2}}{T^{3}\Gamma \left(4-\alpha \right)}t^{3-\alpha }+\frac{{\left(\lambda +\mu \right)}^{2}}{T^{2}}t^{2}, & \text{otherwise};\\ \frac{2L_{0}C\left(\lambda +\mu \right)+C\sigma^{2}}{T^{2}\Gamma \left(3-\alpha \right)}t^{2-\alpha }+\frac{2C^{2}{\left(\lambda +\mu \right)}^{2}}{T^{4}\Gamma \left(5-2\alpha \right)}, & \text{for}\;\frac{C}{T}\gg 1. \end{array}\right.$$

Then we have

(21) $$\langle \Delta L^{2}\left(t\right)\rangle =\left\{\begin{array}{ll} \frac{\sigma^{2}}{T}t, & \text{for}\;\frac{C}{T}\ll 1;\\ \frac{\sigma^{2}}{T}t+\frac{C\sigma^{2}}{T^{2}\Gamma \left(3-\alpha \right)}t^{2-\alpha }+\frac{2C{\left(\lambda +\mu \right)}^{2}}{T^{3}\Gamma \left(4-\alpha \right)}t^{3-\alpha } & \\ +\left[\frac{2}{\Gamma \left(5-2\alpha \right)}-\frac{1}{\Gamma^{2}\left(3-\alpha \right)}\right]\frac{C^{2}{\left(\lambda +\mu \right)}^{2}}{T^{4}}t^{4-2\alpha }, & \text{otherwise};\\ \left[\frac{2}{\Gamma \left(5-2\alpha \right)}-\frac{1}{\Gamma^{2}\left(3-\alpha \right)}\right]\frac{C^{2}{\left(\lambda +\mu \right)}^{2}}{T^{4}}t^{4-2\alpha } & \\ +\frac{C\sigma^{2}}{T^{2}\Gamma \left(3-\alpha \right)}t^{2-\alpha }, & \text{for}\;\frac{C}{T}\gg 1, \end{array}\right.$$

which has the asymptotic expression

(22) $$\langle \Delta L^{2}\left(t\right)\rangle =\left\{\begin{array}{ll} \frac{\sigma^{2}}{T}t, & \text{for}\;\frac{C}{T}\ll 1;\\ \frac{2C{\left(\lambda +\mu \right)}^{2}}{T^{3}\Gamma \left(4-\alpha \right)}t^{3-\alpha }, & \text{otherwise};\\ \left[\frac{2}{\Gamma \left(5-2\alpha \right)}-\frac{1}{\Gamma^{2}\left(3-\alpha \right)}\right]\frac{C^{2}{\left(\lambda +\mu \right)}^{2}}{T^{4}}t^{4-2\alpha }, & \text{for}\;\frac{C}{T}\gg 1, \end{array}\right.$$

When C/T≪1, the EAMSD is proportional to t, which is the normal diffusion process (see Figure 3C). When C/T≫1, the EAMSD is proportional to $t^{4-2\alpha }$, which indicates that the TS process is the anomalous diffusion process (see Figure 3E). In other cases, the EAMSD is proportional to $t^{3-\alpha }$, which shows that the TS process is the superdiffusion process (see Figure 3D).

## 3 THE FEYNMAN–KAC EQUATION OF TS PROCESS

We derive the Feynman–Kac equation describing the functional distribution of the TS process modeled by the CTRW with the waiting time obeying the tempered power law distribution [35, 36].

Tempering anomalous diffusion is often used to describe the transition between normal diffusion and anomalous diffusion. The tempering power law distribution is e^−*ξt*^ *ω*(*t*), where *ξ* is the tempering parameter. One can see that when *t* is small, the exponent *e* ^−*ξt*^ *ω*(*t*) has no effect on *ω*(*t*); while for large *t*, the exponent *e* ^−*ξt*^ has an effect on *ω*(*t*). This fully indicates that the use of tempering can well describe anomalous diffusion in a practical situation. The results in Section 2 show that by taking different values of *C*/*T*, the evolution of the TS process is between normal diffusion and anomalous one, which is very similar to tempering the anomalous diffusion of particles [37]. Therefore, we will link the evolution of the TS process with tempered anomalous diffusion and revise the waiting time distribution in the CTRW model from the power law distribution *ω*(*t*) to the tempered power law distribution *ω*(*t*, *ξ*). Ref. [38] gives the Laplace transform form of *ω*(*t*, *ξ*), that is,

(23) $$\hat{\omega }\left(s,\xi \right)=\int_{0}^{\infty }e^{-st}\omega \left(t,\xi \right)dt=e^{\hat{\phi }\left(s,\xi \right)},$$

where $\hat{\phi }\left(s,\xi \right)=-B_{\gamma }{\left(s+\xi \right)}^{\gamma }+B_{\gamma }\xi^{\gamma }$. Then

(24) $$\hat{\omega }\left(s,\xi \right)=e^{-B_{\gamma }{\left(s+\xi \right)}^{\gamma }+B_{\gamma }\xi^{\gamma }}∼1-B_{\gamma }{\left(s+\xi \right)}^{\gamma }+B_{\gamma }\xi^{\gamma }$$

for small *s* and *ξ*.

### 3.1 Forward Feynman–Kac equation

Define $A=\int_{0}^{t}U\left[L\left(\tau \right)\right]d\tau ,$ where *U*(*L*) is a given function and *L*(*τ*) is a process of TS length. Let *G*(*L*, *A*, *t*) represent the joint PDF of *L* and *A* at time *t*. Then the integral equation for *G*(*L*, *A*, *t*) can be built as [39]

(25) $$\begin{array}{c} G\left(L,A,t\right)=\int_{0}^{t}W\left(\tau ,\xi \right)Y\left[L,A-\tau U\left(L\right),t-\tau \right]d\tau , \end{array}$$

where $W\left(\tau ,\xi \right)=\int_{\tau }^{+\infty }\omega \left(\tau^{\prime },\xi \right)d\tau^{\prime }$ is the probability that TS length does not change at the time interval [0, *t*]. There are two reasons why the shortened length does not change. On the one hand, the observation time is relatively short, and the cells do not go through a complete cell cycle. On the other hand, the cells are already senescent at the time of observation and could no longer divide. Denote *Y*(*L*, *A*, *t*)d*t* as the probability that the TS length “jumps” into (*L*, *A*) in [*t*, *t* + d*t*]. Then, we also perform Laplace transform w.r.t. *t* and Fourier transforms w.r.t. *A* and *L*, resulting in the following:

(26) $$\begin{array}{c} \tilde{\hat{G}}\left(k,p,s\right)=\hat{W}\left[s-ipU\left(-i\frac{\partial }{\partial k}\right),\xi \right]Y\left(k,p,s\right)=\frac{1-\hat{\omega }\left[s-ipU\left(-i\frac{\partial }{\partial k}\right),\xi \right]}{s-ipU\left(-i\frac{\partial }{\partial k}\right)}\tilde{\hat{Y}}\left(k,p,s\right). \end{array}$$

Here, we use $\hat{W}\left(s,\xi \right)=\int_{0}^{+\infty }e^{-st}W\left(t,\xi \right)dt=\left[1-\hat{\omega }\left(s,\xi \right)\right]/s.$ According to the evolution of the process, there exists

$$Y\left(L,A,t\right)=\int_{0}^{t}\omega \left(t,\xi \right)\left\{\int_{-\infty }^{+\infty }\varphi \left(y\right)Y\left[L-y,A-\tau U\left(L-y\right),t-\tau \right]dy\right\}d\tau +\delta \left(L\right)\delta \left(A\right)\delta \left(t\right);$$

we take the initial condition G(L,A,t)=δ(A)δ(L)δ(t), and perform the Fourier transform w.r.t. *A* and the Laplace transform w.r.t. *t*. Then, we obtain the following:

(27) $$\begin{array}{c} \tilde{\hat{Y}}\left(k,p,s\right)=\frac{1}{1-\tilde{\varphi }\left(k\right)\hat{\omega }\left[s-ipU\left(-i\frac{\partial }{\partial k}\right),\xi \right]}. \end{array}$$

What we need to note is the order of $\tilde{\varphi }\left(k\right)$ and $\hat{\omega }\left[s-ipU\left(-i\frac{\partial }{\partial k}\right),\xi \right]$ cannot be switched. Then we get the following:

(28) $$\begin{array}{c} \tilde{\hat{G}}\left(k,p,s\right)=\frac{1-\hat{\omega }\left[s-ipU\left(-i\frac{\partial }{\partial k}\right),\xi \right]}{s-ipU\left(-i\frac{\partial }{\partial k}\right)}\times \frac{1}{1-\tilde{\varphi }\left(k\right)\hat{\omega }\left[s-ipU\left(-i\frac{\partial }{\partial k}\right),\xi \right]}. \end{array}$$

Substituting $\tilde{\varphi }\left(k\right)$ and $\hat{\omega }\left(s,\xi \right)$ into Equation (28), we have

$$\left[\frac{-ik\left(\lambda +\mu \right)\left(1+B_{\gamma }\xi^{\gamma }\right)}{B_{\gamma }}+\frac{\sigma^{2}k^{2}}{2B_{\gamma }}-\xi^{\gamma }\right]{\left[\xi +s-ipU\left(-i\frac{\partial }{\partial k}\right)\right]}^{1-\gamma }\tilde{\hat{G}}\left(k,p,s\right)$$

(29) $$\begin{array}{c} +\left[\xi +s-ipU\left(-i\frac{\partial }{\partial k}\right)\right]\tilde{\hat{G}}\left(k,p,s\right)=\frac{e^{ikL_{0}}\left\{\xi -\xi^{\gamma }{\left[\xi +s-ipU\left(-i\frac{\partial }{\partial k}\right)\right]}^{1-\gamma }\right\}}{s-ipU\left(-i\frac{\partial }{\partial k}\right)}+1. \end{array}$$

Now, the forward Feynman–Kac equation can be obtained by taking the inverse transformation

(30) ∂∂tG(L,p,t)=−(λ+μ)1+BγξγBγ∂∂L+σ2k22Bγ∂2∂L2+ξγD0t1−γ,ξG(L,p,t)−[ξ−ipU(L)]G(L,p,t)+ξ−ξγD0t1−γ,ξeipU(L)tδ(L),

where the operator Dt1−γ,ξ0 is defined as follows:

(31) D0t1−γ,ξG(L,p,t)=1Γ(α)ξ−ipU(L)+∂∂t∫0te−(t−τ)(ξ−ipU(L))(t−τ)1−αG(L,p,τ)dτ.

### 3.2 Backward Feynman–Kac equation

In some practical applications, we are only interested in the distribution of the functional *A*. Then, it is natural to wonder if the distribution of *A* can be obtained directly by a simpler method, which is the content of the backward Feynman–Kac equation. Here, it is easy to get the equation of $G_{L_{0}}\left(A,t\right)$ (the PDF of *A* at time *t*), given that the process has started at *L*_0_.

Considering the initial condition $G_{L_{0}}\left(A,t=0\right)=\delta \left(A\right)$ and according to the CTRW model, the integral equation for $G_{L_{0}}\left(p,t\right)$ can be built as follows:

(32) $$G_{L_{0}}\left(A,t\right)=\int_{0}^{t}\omega \left(\tau ,\xi \right)\int_{-\infty }^{+\infty }G_{L_{0}+y}\left[A-\tau U\left(L_{0}\right),t-\tau \right]\times \varphi \left(y\right)dyd\tau +W\left(t,\xi \right)\delta \left[A-tU\left(L_{0}\right)\right].$$

By taking the Laplace transform *t* → *s* and the Fourier transform *A* → *p*, *L*_0_ → *k*_0_, we obtain

(33) $$s\;{\hat{\tilde{G}}}_{k_{0}}\left(p,s\right)-\delta \left(k_{0}\right)={\left[\xi +s-ipU\left(-i\frac{\partial }{\partial k_{0}}\right)\right]}^{1-\gamma }\times \left[-\frac{\left(\lambda +\mu \right)\left(1+B_{\gamma }\xi^{\gamma }\right)}{B_{\gamma }}ik_{0}-\frac{\sigma^{2}}{2B_{\gamma }}k_{0}^{2}+\xi^{\gamma }\right]\times {\hat{\tilde{G}}}_{k_{0}}\left(p,s\right)-\left[\xi -ipU\left(-i\frac{\partial }{\partial k_{0}}\right)\right]{\hat{\tilde{G}}}_{k_{0}}+\frac{\xi -{\left[\xi +s-ipU\left(-i\frac{\partial }{\partial k_{0}}\right)\right]}^{1-\gamma }\xi^{\gamma }\;}{s-ipU\left(-i\frac{\partial }{\partial k_{0}}\right)}\delta \left(k_{0}\right).$$

Applying the inverse transformation, there exists

(34) ∂∂tGL0(p,t)=D0t1−γ,ξ(λ+μ)1+BγξγBγ∂∂L0+σ22Bγ∂2∂L02+ξγGL0(p,t)−ξ−ipUL0GL0(p,t)+ξ−ξγD0t1−γ,ξeipUL0t.

It is important to note that whether we derive the forward or backward equation, we always do the Fourier transform with respect to the functional *A*. However, if *A* is positive, we can also choose to use the Laplace transform, and the equation formally becomes

∂∂tG(L,p,t)=−(λ+μ)1+BγξγBγ∂∂L0+σ22Bγ∂2∂L2+ξγDt1−γ,ξ0G(L,p,t)

(35) −[ξ+ipU(L)]G(L,p,t)+ξ−ξγD0t1−γ,ξeipU(L)tδ(L),

and

(36) ∂∂tGL0(p,t)=D0t1−γ,ξ(λ+μ)1+BγξγBγ∂∂L0+σ22Bγ∂2∂L02+ξγGL0(p,t)−ξ+ipUL0GL0(p,t)+ξ−ξγD0t1−γ,ξeipUL0t.

## 4 APPLICATION OF FEYNMAN–KAC EQUATION

We derive the Feynman–Kac equations, which describe the dynamics of the TS process from two different perspectives. In this section, we will focus on the distribution of functional *A* for TS length. For convenience, we start directly from the backward Feynman–Kac equation, the solution of which is the PDF of the functional *A* in the frequent domain. We know that when the shortest telomere length in the cell is below the critical value *l*_*c*_, the cell division will be limited, and then gradually senescent. Here, we will pay attention to two types of biometric statistics related to *l*_*c*_—occupation time and first passage time [40, 41].

### 4.1 Occupation time

The research studies on occupation time have a wide range of applications in materials science, biophysics, complex networks, finance, etc. [42, 43, 44]. For example, in materials science, studying the occupation time of a material’s surface helps to understand processes such as chemical reactions, surface adsorption, and film growth. In biophysics, studying the occupation time of aggregates within cells can reveal the fundamental laws of cell transport and metabolism. For the random particle swarm systems, the study of occupied time can help us understand the dynamic behavior of local aggregation and diffusion in the system.

**FIGURE 4:**
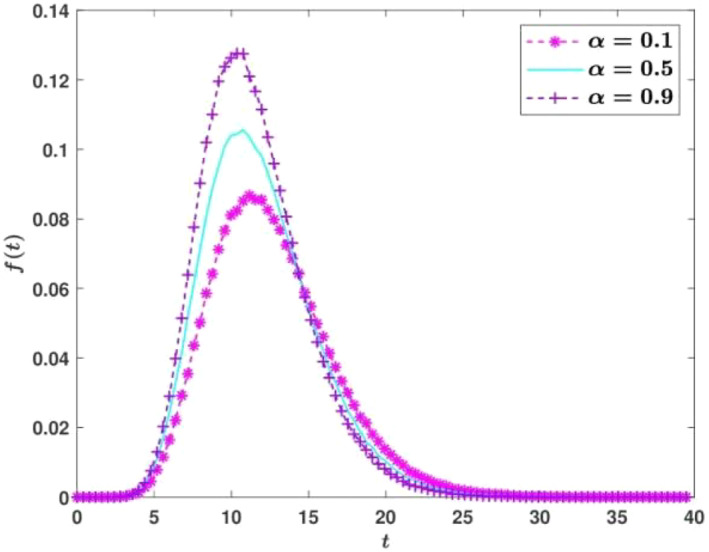
The behavior of *J*(*t*) with respect to different parameters *α*, where *ξ* = 0.1, *λ* = 155, *μ* = 60, *σ* = 1, and the shortened length interval is [0, 7000].

The occupation time in this paper is the total time for a telomere to shorten the length between [0, *l*_0_ − *l*_*c*_] in the observation time [0, *t*], which can be defined as the following:

(37) $$T^{+}=A=\int_{0}^{t}U\left[L\left(\tau \right)\right]\text{d}\tau ,$$

where

(38) $$\begin{array}{c} U\left(L\right)=\left\{\begin{array}{c} 1,L\in \left[0,l_{0}\;-\;l_{c}\right];\\ 0,L\notin \left[0,l_{0}\;-\;l_{c}\right], \end{array}\right. \end{array}$$

in Equation (36). The PDF *J*(*t*) of occupation time can be obtained by solving the backward Feynman–Kac equation (36) (or performing Monte Carlo simulation; see Figure 4).

Studying the total time of TS can provide a deeper understanding of the mechanism of cell aging. Meanwhile, TS is closely related to the occurrence and progression of a variety of diseases, including cancer, cardiovascular diseases, nervous system diseases, etc. It can provide important clues, new ideas, and strategies for disease prevention and treatment, and an important reference for finding new methods and drugs for antiaging. On the whole, studying the occupation time of TS processes can improve the understanding of cellular aging, disease occurrence and progression, and antiaging research.

From Figure 4, one can note that J(t) gradually increases to a peak value with the increase of time and finally decreases to 0. In fact, J(t) also represents a concentration of cells in life whose TS length is in $\left[0,l_{0}\;-\;l_{c}\right]$ at time t. So, from the figure, one can clearly see how long most of the cells’ telomeres reach $l_{c}$.

### 4.2 First passage time

In statistical physics, the first passage time is the time that a particle takes to arrive at or the passage of a particular location for the first time. The study of first passage time plays an important role in understanding and describing a variety of physical phenomena and real‐world systems, including the areas of diffusion, random walks, movement in random media, and financial market fluctuations [40, 41].

Here, the physical quantity described by the first passage time is the time taken by the TS length to reach the critical value *l*_0_ − *l*_*c*_ for the first time *T*_*f*_, which can be defined as follows:

(39) $$T_{f}=\underset{\tau \geq 0}{\inf}\left\{\tau :L\left(\tau \right)\geq \left(l_{0}\;-\;l_{c}\right)\right\},$$

It can reflect the time scale on which cells begin to become senescent cells. Let *T* ^+^ represent the occupation time where the TS length is greater than *l*_0_ − *l*_*c*_. Then the distribution of the first passage time can be described as follows:

$$P_{r}\left\{T_{f}>t\right\}=P_{r}\left\{\max_{0\leq \tau \leq t}L\left(\tau \right)<\left(l_{0}\;-\;l_{c}\right)\right\}=P_{r}\left\{T^{+}=0\right\}$$

(40) $$=\lim_{p\to \infty }G_{L_{0}}\left(p,t\right).$$

The PDF of the first passage time *f*(*t*) satisfies

(41) $$f\left(t\right)=\frac{\partial }{\partial t}\left(P_{r}\left\{T_{f}\leq t\right\}\right)=\frac{\partial }{\partial t}\left(1-P_{r}\left\{T_{f}>t\;\right\}\right)\;.$$

It is worth noting that the probability that the TS length does not reach *l*_0_ − *l*_*c*_ during the observation time [0, *t*] is equal to the probability that the maximum length of TS is less than *l*_0_ − *l*_*c*_ during the observation time. So, *f*(*t*) should be equal to *J*(*t*) in this context.

## 5 CONCLUSION

Regarded by scientists as the clock of life, telomeres gradually shorten during cell division, and TS is thought to be an important factor in cellular aging and disease. To study the TS process, many mathematical models have been developed to try to reveal the microscopic mechanism of the TS process from a random perspective. In this paper, we link the TS process with the nonstationary, aging di used molecular view, and for the first time link the TS process with nonequilibrium statistical physics and conduct a theoretical modeling study on the TS process from the perspective of random processes. We have built the CTRW model to describe the TS process. Based on the CTRW model, two types of macroscopic equations, that is, Fokker–Planck and Feynman–Kac equations have been derived with some applications, including calculating moments, occupation time, and first passage time. In the future work, we will build the model involving the action of telomerase and more practical/experimental data.

## AUTHOR CONTRIBUTIONS

**Panpan Han**: Formal analysis; investigation; methodology; writing—original draft. **Yang Zhou**: Formal analysis; investigation; writing—original draft. **Weihua Deng**: Conceptualization; funding acquisition; investigation; methodology; project administration; writing—review and editing.

## CONFLICT OF INTEREST STATEMENT

The authors declare no conflicts of interest.

## ETHICS STATEMENT

This article does not contain any studies with human or animal subjects performed by any of the authors.

## Data Availability

The data are available from the corresponding author on reasonable request.
